# Exosomes in hypoxia: generation, secretion, and physiological roles in cancer progression

**DOI:** 10.3389/fimmu.2025.1537313

**Published:** 2025-06-04

**Authors:** Yufeng Mu, Mengrui Yang, Jingyang Liu, Yan Yao, Huimin Sun, Jing Zhuang

**Affiliations:** ^1^ First School of Clinical Medicine, Shandong University of Traditional Chinese Medicine, Jinan, China; ^2^ Antitumor Omics Laboratory of Traditional Chinese Medicine, Weifang Traditional Chinese Hospital, Weifang, China; ^3^ State Key Laboratory of Quality Research in Chinese Medicine, and Faculty of Chinese Medicine, Macau University of Science and Technology, Macao, Macao SAR, China; ^4^ College of Traditional Chinese Medicine, Shandong Second Medical University, Weifang, China; ^5^ Department of Pathology, The First Clinical Medical College of Shandong Second Medical University, Weifang people’s Hospital, Weifang, China; ^6^ Department of Oncology, Weifang Traditional Chinese Hospital, Weifang, China

**Keywords:** hypoxia, exosomes, TME (tumor microenvironment), tumor, oncogenic cargoes

## Abstract

The hypoxic microenvironment represents a universal hallmark feature of most solid tumors, profoundly shaping cancer progression through multifaceted mechanisms. Acting as nanoscale molecular envoys, exosomes transport oncogenic cargoes (including non-coding RNAs, mutated proteins, and metabolites) to reprogram stromal cells, prime pre-metastatic niches, and establish tumor-host metabolic symbiosis. Their lipid bilayer architecture ensures the protection of labile hypoxia-responsive factors, positioning them as critical amplifiers of intercellular crosstalk within the tumor microenvironment. Despite significant advances, critical gaps persist in understanding the spatiotemporal regulation of exosomal release under hypoxia, particularly the organ-specific variations in hypoxic exosome signatures revealed by single-vesicle analyses. This review synthesizes recent advances in the intricate interplay between hypoxia and exosomes, emphasizing hypoxia-related signaling pathways that directly modulate exosome biogenesis and indirectly activate hypoxia-associated microenvironmental remodeling, alongside their distinct regulatory effects on exosomal cargo composition. Furthermore, it delineates the pivotal role of hypoxia-specific exosomes in driving cancer malignancy, including metastatic dissemination, immune evasion, and therapy resistance. By integrating molecular mechanisms with clinically actionable insights, this work establishes a translational framework for targeting the hypoxic exosome network in precision oncology, offering strategic references for biomarker discovery and therapeutic development.

## 1 Introduction

Hypoxia, a hallmark of cancer, is present in 90% of solid tumors (1, 2). Existing clinical data obtained through polarographic measurement of tumor partial oxygen pressure (pO2) demonstrate significantly low values (<10 mmHg) across multiple tumor types, including pancreatic cancer, head and neck carcinoma, cervical carcinoma, and melanoma (3–6). Intratumoral hypoxia has been closely associated with disease progression and reduced disease-free survival rates in several malignancies, notably prostate cancer, cervical cancer, and head and neck squamous cell carcinoma (HNSCC) (4–7). Mechanistic studies reveal that hypoxic microenvironments primarily drive oncogenesis through transcriptional reprogramming of genes regulating metabolism and other cellular processes. Furthermore, hypoxia signaling interacts with diverse cellular pathways to enhance malignant behaviors of cancer cells, including proliferation, migration, invasion, and angiogenesis, while concurrently compromising therapeutic efficacy (8). These multidimensional effects position hypoxia as a critical determinant of tumor evolution and treatment resistance.

Cancer research has been investigating various novel molecular mechanisms associated with cancer to identify more effective therapeutic strategies. In recent years, significant progress has been made in understanding the tumor microenvironment (TME). Studies have shown that the secretion and loading of functional cargo molecules within exosomes, which serve as signaling platforms for intercellular communication, are altered in the tumor microenvironment, particularly under hypoxic conditions (9). These changes influence tumor initiation and progression. Exosomes are a crucial subset of extracellular vesicles (EVs), characterized by their smaller diameter compared to other EV components, allowing them to stably enter the extracellular space. Due to their lipid bilayer structure, natural stability, and ability to traverse multiple biological and physical barriers even in harsh TME conditions, exosomes are considered efficient natural cargo transporters. Additionally, surface molecules on exosomes enable selective targeting of recipient cells, thereby ensuring precise delivery of cargo to specific cells (10). Consequently, exosomes play a critical role in cell-to-cell information transfer and are closely linked to cancer growth, metastasis, and treatment resistance.

Some researchers have proposed a potential link between the significantly higher exosome production and secretion by tumor cells compared to normal tissue cells and the hypoxic conditions prevalent within the TME (11). This association may be attributed to the activation of specific hypoxia-induced signaling pathways and the rapid proliferation of tumors under hypoxic conditions, which intensifies and complicates intercellular communication, thereby necessitating increased exosome production for effective cell signaling. Although research on exosomes is advancing, the unique internal composition, biosynthetic pathways, and cellular uptake mechanisms of hypoxia-related exosomes relative to normoxic exosomes remain underexplored. This review aims to summarize the effects of exosomes in hypoxic microenvironments, elucidate the mechanisms governing exosome production in hypoxic conditions, and investigate their regulatory functions in tumors. Additionally, it seeks to explore potential molecular mechanisms and interactions, providing insights for researchers and clinicians to investigate the clinical applications of exosomes.

## 2 Hypoxia-mediated regulation of tumor exosomes

### 2.1 Hypoxia-induced production of tumor-associated exosomes

The production of exosomes is not strictly programmed but is influenced by a multitude of factors, such as cellular state and external stimuli, leading to variations in the composition and biological functions of exosomes derived from different sources.

**Figure 1:**
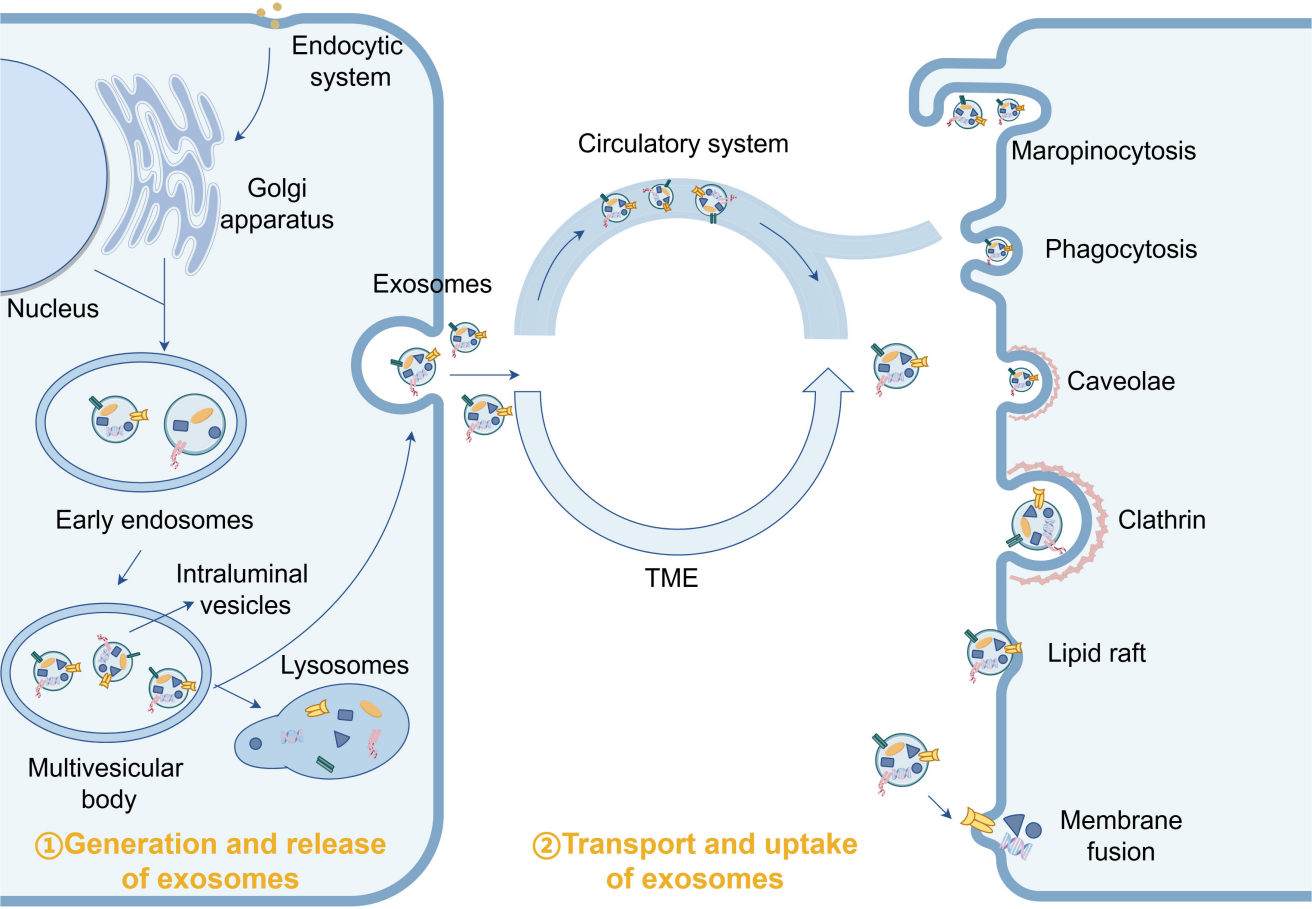
Under normal physiological conditions, exosomes participate in the maintenance of tissue homeostasis by delivering “cargo”.

Exosome biogenesis initiates with endocytosis at the cell membrane surface, forming early endosomes that undergo cargo loading and transform into multivesicular bodies (MVBs) containing intraluminal vesicles (ILVs). The biosynthesis of MVBs is critical for exosome formation. Subsequently, plasma membrane budding generates cup-shaped structures that further develop into early sorting endosomes (ESEs). The trans-Golgi network and endoplasmic reticulum contribute to ESE formation. ESEs mature into late sorting endosomes, followed by invagination of the internal limiting membrane, resulting in the formation of multiple ILVs within MVBs. Consequently, MVBs encapsulate these ILVs, which will eventually become exosomes. Finally, MVBs either fuse with the plasma membrane to release ILVs as exosomes into the extracellular space or fuse with lysosomes or autophagosomes for degradation and metabolism (**Figure 1**) (12, 13).

Under non-hypoxic conditions, multiple mechanisms for MVB generation have been proposed. These include the ESCRT-dependent pathway, specific lipid molecules, and tetraspanin family proteins (14). In the classical ESCRT-dependent pathway, ubiquitinated receptor proteins in endosomal membranes are recognized and recruited, followed by vesicle budding and deubiquitination to form the final cargo proteins. The invagination of the vesicles leads to their contraction and separation from the membrane, ultimately producing MVBs (15–17). The mechanism by which hypoxia mediates MVB production remains unclear; however, it is well established that hypoxia activates multiple signaling pathways that stimulate accelerated exosome secretion. These pathways include hypoxia-inducible factor (HIF), phosphoinositide 3-kinase (PI3K)-Akt, Wnt/β-catenin, mitogen-activated protein kinase (MAPK), Rab GTPases, nuclear factor-κB (NF-κB), and NADPH oxidase (NOX). All these signaling pathways are thought to be involved in exosome synthesis (18–20). Wang et al. found that HIF expressed by hypoxic cells triggered the expression and transcription of the gene encoding the small GTPase RAB22A, leading to enhanced shedding of microvesicles (MVs) (21). Additionally, hypoxia has been shown to induce the expression of Rho-associated protein kinase (ROCK) (22), which plays a role in MV biogenesis in various tumor cell types (23). These studies reveal that hypoxia affects exosome synthesis and release by modulating the expression of signaling pathways, which will be discussed in detail later.

### 2.2 Uptake of tumor-associated exosomes induced by hypoxia

Under normal physiological conditions, ligands on the cell membrane of recipient cells interact with surface molecules on exosomes to facilitate specific exosome uptake. For instance, tetraspanins CD9 and CD81 specifically enhance exosome internalization in certain cell types (24). Research has demonstrated that while exosome uptake is not strictly cell type-specific, the efficiency of uptake varies among different cell types. Peritoneal exudate cells exhibit the highest uptake of exosomes derived from pancreatic cancer, whereas granulocytes show the lowest uptake (25). It can be inferred that there exists a specific targeting transfer function between recipient and donor cells, which is activated only when the ligand-receptor interaction is precise (26). In breast cancer cells, glycoproteins enriched with high mannose or NeuAcα2,3/6 structures are secreted, leading to preferential targeting of certain cell types (27). Conversely, glioblastoma-derived exosomes, which are enriched in phosphatidylethanolamine, selectively target glioblastoma, fibrosarcoma, and breast cancer cells (28). Exosomes derived from lymph node stromal cells expressing Tspan8-α4 complexes are more readily taken up by endothelial and pancreatic cells compared to their parental lymph node stromal cells (29).

Under normal physiological conditions, the cell membrane of recipient cells interacts with surface molecules on exosomes to facilitate exosome uptake. For example, Tetraspanins CD9 and CD81 specifically promote the internalization of exosomes by certain cell types (24). Studies have demonstrated that exosome uptake is not cell type-specific; however, there are variations in uptake efficiency among different cell types. Peritoneal exudative cells exhibit the highest uptake of exosomes derived from pancreatic adenocarcinoma; granulocytes display the lowest uptake (25). We can assume that there is a specific targeting transfer function between recipient cells and donor cells, but only when the ligand and receptor are correctly combined (26). Therefore, the specificity of exosomes relies on the interaction between recipient cell ligands and molecular complexes to which exosomes adhere. Breast cancer cells secrete glycoproteins rich in high mannose or NeuAcα2,3/6 constructs, specifically targeting certain cell types (27). In contrast, glioblastoma-derived exosomes rich in phosphatidyleglycolamine preferentially target glioblastoma, fibrosarcoma, and breast cancer cells (28). Lymph node stromal cell-derived exosomes expressing Tspan8-α4 complex are more likely to be taken up by endothelial cells and pancreatic cells than they are to be taken up by parental lymph node stromal cells (29).

On this basis, the addition of hypoxia factors significantly impacts the transport of specific proteins to the cell surface. Yoon et al. observed that hypoxia promotes the translocation of vesicles containing α6 integrin from the perinuclear region to the plasma membrane, thereby increasing the surface expression of integrin α6β4 via a Rab11-dependent pathway (30). This finding suggests that hypoxia may influence exosome uptake by modulating integrin α6β4. Menard et al. reported that the uptake of major lipoproteins, including high-density lipoproteins (HDL), low-density lipoproteins (LDL), and very low-density lipoproteins (VLDL), is enhanced under hypoxic conditions, with the initial increased uptake likely mediated by heparan sulfate proteoglycans (HSPGs) (31). Given that extracellular vesicles (EVs) directly regulate HSPG raft endocytosis, EV internalization is augmented under hypoxic conditions (32). These observations collectively support the hypothesis that hypoxia enhances the uptake of exosomes by cancer cells, thereby improving the efficiency of tumor exosome uptake by tumor parent cells.

In this process, several researchers have observed that exosomes produced under hypoxic conditions exhibit reduced size, facilitating faster internalization compared to those generated under normoxic conditions. This characteristic enables them to more readily cross physiological barriers, reach other cells, and effectively transport to metastatic sites via the bloodstream (11, 33). Ye et al. noted that the bending modulus of small extracellular vesicles (sEVs) decreases with increasing malignancy, while stiffness and osmolality increase with malignancy but decrease with sEV size (34). These observations suggest an inverse correlation between exosome size and tumor malignancy; however, the underlying mechanisms require further investigation.

### 2.3 Alterations in exosome composition during hypoxia

Exosomes transport a diverse array of cargos, including DNA, mRNA, miRNA, proteins, lipids, metabolites, and other molecules. Under hypoxic conditions, exosomes selectively enrich tumor-promoting factors, resulting in higher concentrations of these components compared to non-hypoxic conditions. This enrichment facilitates tumor progression and development while also presenting potential targets for tumor diagnosis and therapy. In summary, hypoxia not only modifies exosomal cargo but also increases exosome heterogeneity.

#### 2.3.1 Protein

The protein composition of exosomes is highly diverse, and the mechanisms governing protein heterogeneity in hypoxic exosomes are increasingly being elucidated. There appears to be a positive correlation between the abundance of proteins in cancer cells and their presence in exosomes. The increased abundance of specific proteins in certain cell types leads to their preferential incorporation into exosomes during biogenesis (11). However, this mechanism alone does not fully explain the protein loading on exosomes. Zhang et al. demonstrated that cargo can be selectively packaged into exosomes (35), although the underlying mechanism remains unclear. It is known, however, that hypoxia influences molecules involved in protein loading, thereby altering both the type and quantity of proteins in exosomes.Such as, The non-small cell lung cancer A549 cell group exhibits upregulation of 130 exosomal proteins and downregulation of 129 exosomal proteins under hypoxia (36). Moreover, different proteins can endow exosomes with different functions. HIF1-α is selectively loaded into exosomes through its KFERQ-like motif and Lysosome-associated membrane protein 2(LAMP2A) in endosomes, shifting the transcriptional activity of HIF1-α from hypoxic to normoxic cells (37) and further promoting the formation and enhancement of a hypoxic microenvironment. In addition to associated protein signals, Wnt4 exosomes enriched under hypoxia are also thought to promote metastasis in normoxic cells (38). In prostate cancer, many proteins were observed in hypoxia (160 proteins) and normoxia (62 proteins), primarily associated with remodeling the epithelial adhesion junction pathway (39). Selective elevation of lysyl oxidase (LOX), thrombospondin-1(TSP1), VEGF, and ADAM Metallopeptidase With Thrombospondin Type 1 Motif 1(ADAMTS1) in hypoxic exosomes in glioblastoma cells; these exosomes exhibited increased angiogenesis-related parameters compared to those exhibited under normoxia (40). Glioblastoma cells also express increased Cx43 within exosomes under hypoxia, and exosome Cx43 contributes to glioma angiogenesis (41).

#### 2.3.2 Nucleic acids

Exosomes carry diverse RNA and DNA sequences. However, a review of the literature reveals that studies on hypoxic cancer cell-derived exosomal DNA are notably scarce. This scarcity may be attributed to the active secretion of extracellular DNA through an autophagy- and multivesicular endosome-dependent mechanism that operates independently of exosomes (42). Analysis of miRNA profiles in melanoma exosomes under normoxic and hypoxic conditions demonstrated that three miRNAs were highly expressed under normoxic conditions, whereas 15 miRNAs showed significant upregulation under hypoxic conditions (43). This evidence further indicates that hypoxic stress modulates the expression of specific miRNAs (44).

In hypoxic gastric cancer cells, microRNA(miR)-301a-3p is enriched and transmitted between gastric cancer cells via exosomes, thereby establishing a synergistic positive feedback loop involving miR-301a-3p/PHD3/HIF-1α to facilitate the proliferation, invasion, migration, and epithelial-mesenchymal transition of gastric cancer cells (45). In bladder cancer 5637 cells, hypoxic exosomes express more lncRNA-UCA1 than normoxic exosomes do; the enriched lncRNA-UCA1 can promote tumor growth and progression by inducing epithelial-mesenchymal transition *in vitro* and *in vivo (* 46). Cellular and exosome miR-155 are significantly upregulated under hypoxia in hepatocellular carcinoma (HCC) cells, and exosome miR-155 may potentially impact the angiogenic activity of HCC (47).

## 3 Hypoxic TME influences the generation and release mechanism of exosomes

Many studies have highlighted the effects of hypoxic exosome production, yet the molecular mechanisms governing exosome loading and secretion under hypoxic conditions remain elusive. Both direct and indirect evidence indicates that a hypoxic microenvironment upregulates exosome biogenesis and influences cargo selection. Additionally, it has been suggested that the impact of hypoxia on exosome characteristics may vary among different cancer cell types, although its overall influence on exosomes is indisputable. Therefore, a comprehensive understanding of the mechanisms underlying exosome production by cancer cells in hypoxic environments will facilitate the advancement of tumor-targeted therapies utilizing exosomes. This review aims to elucidate the mechanisms of exosome production under hypoxic conditions by examining signaling pathways and metabolites.

### 3.1 Signaling pathways activated in response to Hypoxia TME

#### 3.1.1 Activation of the classical signaling pathway HIF

**Figure 2:**
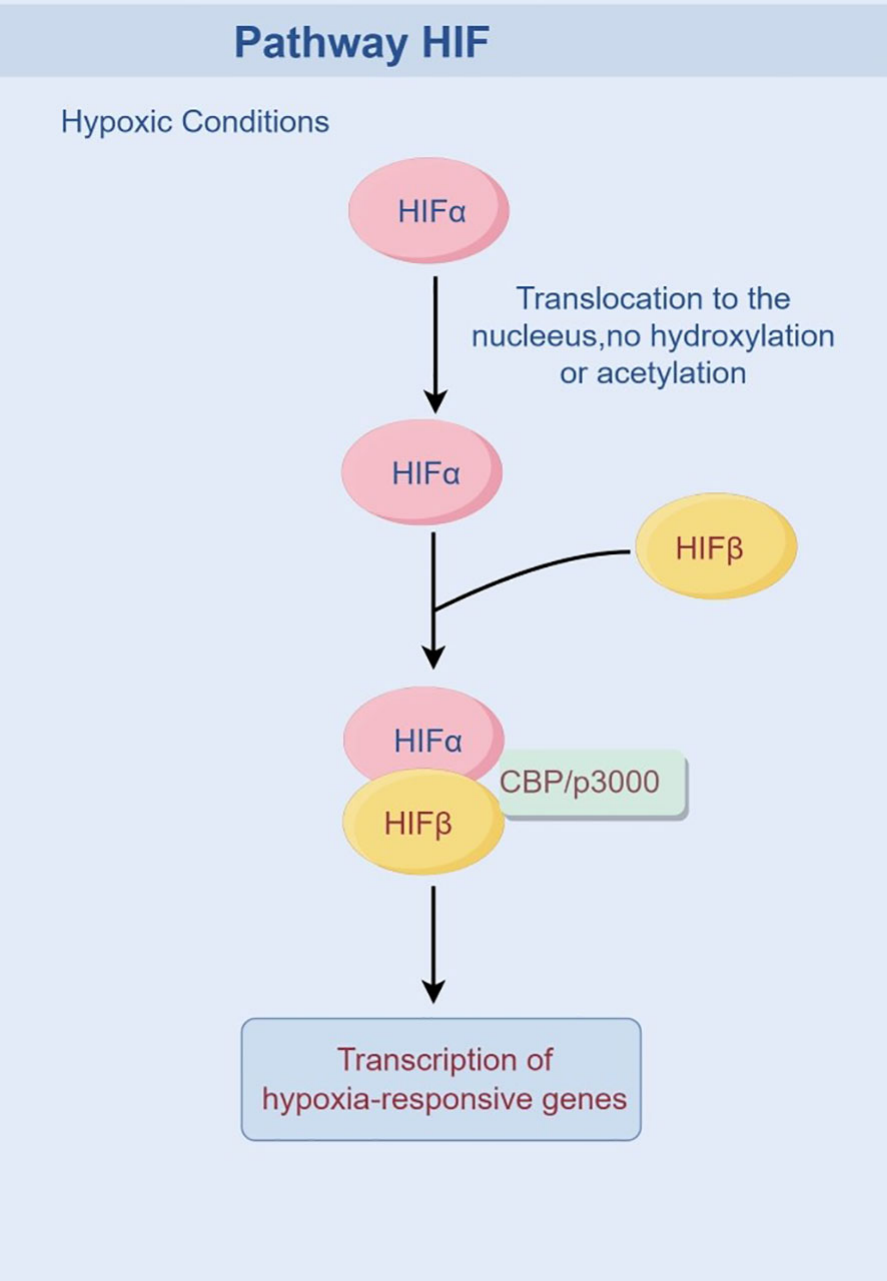
Activated HIF pathway under hypoxic conditions.

HIF is a transcription factor that plays a critical role in cellular adaptation to hypoxia. Activation of the HIF system is a pivotal event in tumor cells under hypoxic conditions, triggering the cellular adaptive response mediated by HIF-1 to coordinate the organism’s response to low oxygen levels. It is now well-established that hypoxia alters the stability of the HIF-1α protein. Under normoxic conditions, prolyl hydroxylase domain proteins (PHD1, 2, or 3) hydroxylate HIF-1α subunits in an O2-, 2-OG-, and iron-dependent manner. This hydroxylation enhances the binding affinity of HIF-1α for pVHL (48), leading to its subsequent ubiquitination by the E3 ubiquitin ligase VHL and degradation via the proteasome pathway. Hydroxylated HIF-1α also inhibits the recruitment of the transcriptional coactivator CBP/p300. In contrast, under hypoxic conditions, PHD activity is inhibited due to reduced oxygen availability, preventing the hydroxylation of HIF-1α. Consequently, the stabilized HIF-1α protein translocates to the nucleus, where it dimerizes with HIF-1β, binds to hypoxia response elements (HREs) in target gene promoters, recruits CBP/p300, and initiates gene transcription (49), thereby upregulating HIF signaling and activating downstream cascades (**Figure 2**).

HIF can modulate the production and composition of exosomes in response to varying signaling inputs, leading to alterations in both the quantity and content of exosomes. King et al. exposed breast cancer cells to moderate hypoxia (1% O_2_) and severe hypoxia (0.1% O_2_) and concluded that the release of exosomes was induced by HIF (50). Gonzalez-King et al. demonstrated that HIF-1α overexpression enhanced exosome secretion under normoxia (51). Interferons (IFN)-induced ISGylation is a negative regulator of HIF-1α, reducing exosome release through lysosomal degradation, suggesting that HIF may participate in exosome secretion by inhibiting the degradation pathway (52, 53). We hypothesize that hypoxia enhances the release of tumor exosomes through activation of the HIF pathway, thereby promoting signal transmission and facilitating rapid tumor expansion. In most HIF-mediated signaling pathways, exosome production increases in a HIF-dependent manner. However, in certain cases of neurobehavioral dysfunction, hypoxia can paradoxically reduce exosome release and impair motor and sensory inhibition (11, 54). Therefore, the regulation of exosome numbers by HIF appears to be bidirectional and influenced by specific microenvironmental demands associated with different diseases.

In the context of exosomal cargo modification, hypoxia-coordinated HIF signaling pathways enhance cellular cargo secretion, thereby promoting tumor survival and development. Oral squamous cell carcinoma cells exposed to hypoxia upregulate microRNA-21 in exosomes through the HIF-1α signaling pathway to modulate the TME (55). Furthermore, proteins are also selectively enriched in exosomes via a HIF1-α-dependent mechanism. HIF-1α depletion in nasopharyngeal carcinoma (NPC) cells leads to reduced MMP-13 in exosomes; in contrast, MMP-13 is overexpressed in exosomes under hypoxia, enhancing tumor cell migration and invasion (56). It is worth mentioning that to healthy cells secrete body still can deliver higher levels of active HIF-1α, which transfer the tumorigenic feature to the new host cells (57). As an essential regulator widely expressed in tumor tissues, HIF-1α acts as a trigger for numerous biological activities and participates in all stages of tumor progression. Future research should focus on elucidating the mechanisms by which HIF-1α inhibits exosome production and subsequently tumor progression.

#### 3.1.2 Additional signaling pathways activated by hypoxia

As previously discussed, hypoxia not only activates HIF but also triggers the activation of other signaling pathways. Despite limited literature on these pathways, both direct and indirect evidence supports the hypothesis that hypoxic exosomes are released via these signaling cascades.

The Rab family proteins belong to the Ras superfamily of small GTPases. Various Rab proteins localize to specific sites on the cytoplasmic side of the plasma membrane and play a role in intracellular vesicle trafficking (58). Rab proteins undergo a transition between two distinct forms: the active form (GTP) and the inactive form (GDP), with effector molecules specifically binding to the active form. Rab5a regulates vesicle formation on the plasma membrane and microtubule-dependent movement of endocytic structures (59, 60). Panigrahi et al. showed Rab5 aggregation around the perinuclear region under hypoxia, possibly contributing to increased exosomes in prostate cancer cells (61). Dorayappan et al. revealed that hypoxia significantly enhanced exosome release in ovarian cancer cells through Rab27a upregulation and Rab7 downregulation (62).

NF-κB is a transcription factor rapidly induced and hyperactivated in various types of cancer (63). The induction mechanism of one of the crucial signaling molecules induced by hypoxia, independent of IKK (inhibitor of NF-κB kinase complex), was proposed in 1994 (64). Hypoxia-induced NF-κB activation differs from the typical activation characterized by IκBα degradatio. However, the direct impact of NF-κB on exosome biogenesis remains unclear. Nonetheless, Yang et al. demonstrated that NF-κB inhibition resulted in alterations to the redox-regulatory enzymes within exosomes derived from the serum of NF-κB knockout mice (65).

Other hypoxic signaling pathway molecules, such as CD9 and CD82, also enhance exosome production upon transfection into HEK 293T cells (66). However, the precise mechanisms by which they regulate exosome release remain to be elucidated.

### 3.2 Hypoxic microenvironment activates oxidative stress

Under hypoxic conditions, the imbalance between reactive oxygen species (ROS) and antioxidants predisposes cells to oxidative damage, disrupting REDOX signaling and homeostasis as well as causing molecular damage. This phenomenon is commonly referred to as oxidative stress (67, 68). Oxidative stress caused by hypoxia can disrupt the intracellular REDOX balance, leading to ROS accumulation and the production of other oxidative by-products, and these oxidators can regulate various intracellular signaling pathways and regulatory mechanisms, thereby affecting exosome biogenesis.

As one of the key responses initiated by tumors, REDOX pathway has been found to directly affect the release of exosomes through post-translational modification of exosome surface proteins (69). In a REDOX dependence mercaptan modification to explore, discover protein thiol REDOX modification may be directly regulate EV release in response to a change in the cell oxidation-reduction environment, act as regulate secrete body release switch (70). Hedlund et al. showed that oxidative stress exacerbated exosome secretion by cancer cells, including leukemia/lymphoma T and B cell lines (71). Atienzar‐Aroca et al. discovered that oxidative stress in retinal pigment epithelial cells enhanced exosome release and promoted endothelial cell angiogenesis (72). In an experiment in which Pt nanoparticles enhanced the release of exosomes from human lung epithelial adenocarcinoma cells, oxidative stress was also found to promote the secretion of exosomes from cancer cells (73). These observations suggest that hypoxia can elevate intracellular oxidative stress levels and impact exosome secretion.

And oxidative stress also affects cargo in exosomes, The redoxin sensitive signal path PI3K/Akt/eNOS regulate secrete body release Angiopoietin 2 (Ang2), and the Ang2 is an important participant of tumor vascular remodeling (74, 75). Maria Eldh et al. also found that exosomes released from mouse mast cells in response to oxidative stress differed in mRNA content (76).

It is worth noting that studies now generally believe that the effects of exosomes and oxidative stress are reciprocal, and while oxidative stress stimulates exosome secretion, exosomes from tumor sources also exacerbate the toxicity of oxidative stress, thus further supporting malignant tumor growth. For instance, exosomes derived from glioblastoma cells augment oxidative stress-induced toxicity by reducing total antioxidant capacity and glutathione (GSH) levels (77). Pancreatic cancer cells overexpressing Vanin-1 (VNN1) secrete cysteamine and exosomes, inducing oxidative stress and thereby exacerbating the dysfunction of paraneoplastic islets (78).

### 3.3 Hypoxia promote acidic microenvironment formation

During cell transformation, the lactate metabolic pathway undergoes reprogramming, causing tumor cells to redirect their original glucose metabolism from efficient oxidative phosphorylation to the less efficient glycolytic pathway, known as the “Warburg effect” (79). Hypoxia enhances the Warburg effect, leading to intracellular accumulation of lactate and bicarbonate and a consequent decrease in pH within the TME. Ban et al. discovered that acidic pH can enhance exosome stability *in vitro*, improving exosome isolation yield (80). Parolini et al. showed that acidic conditions promote exosome release and melanoma cell metastasis (81). Logozz et al. demonstrated that an acidic pH increases exosome release from human cancer cells independent of the original cancer type, and buffering the acidic TME reduces exosome release (82). Hypoxia activates pyruvate kinase isoform PKM2 to stimulate glycolysis and lactate production, shaping an acidic microenvironment (83, 84). PKM2 also enhances exosome secretion (85).

## 4 The promotion of tumor progression is facilitated by exosomes derived from hypoxic tumors

**Figure 3:**
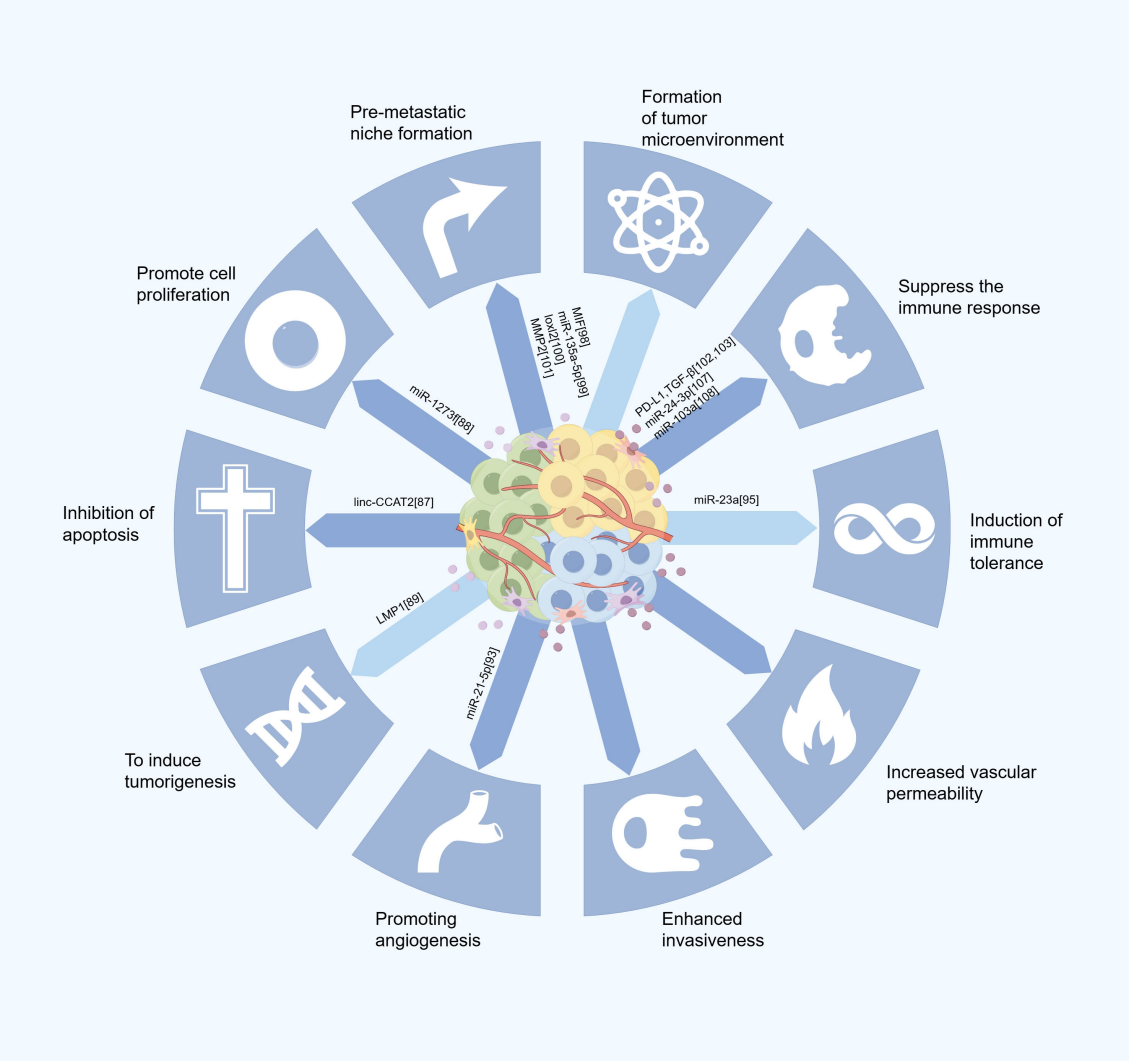
Schematic overview of exosomal modulation tumor microenvironment (TME).

In the normal cellular physiological environment, exosomes facilitate the intercellular transfer of bioactive molecules and coordinate cell growth, proliferation, and apoptosis. In tumor cells, the hypoxic microenvironment alters the biogenesis of exosomes, leading to increased production and release from various tumor cell types. These tumor-derived exosomes interact with immune and stromal cells to promote cancer cell immune evasion, induce immune tolerance, and enhance their survival (**Figure 3**).

### 4.1 Influence on tumor growth

Hypoxia can induce genetic and proteomic alterations in cancer cells, exerting selective pressure that promotes the emergence of a more aggressive phenotype. Notably, hypoxic conditions predominantly occur at the distal end of the invasion front, typically at the tumor periphery (39). Consequently, exosomes serve as critical signaling mediators facilitating intercellular communication between peripheral and central tumor regions. Many studies have shown that exosomes facilitate tumor cell communication within hypoxic microenvironments in various cancers, such as lung (86), prostate (39), breast (21), colorectal (87), and oral (55) cancers.

Exosomes also play a role in inhibiting apoptosis, regulating tumor cell proliferation, and inducing tumorigenesis. In glioma cells, exosomes enriched with linc-CCAT2 are internalized by human umbilical vein endothelial cells (HUVEC), resulting in reduced HUVEC apoptosis under hypoxia through Bcl-2 upregulation and downregulation of Bax and caspase-3 (88). In HCC cells, hypoxia induces increased exosome production, promoting miR-1273f expression in normoxic cells and enhancing malignant phenotype partially by targeting LHX6 downregulation and facilitating the proliferation and malignant transformation of normoxic tumor cells (89). Maja et al. observed that the induction of latent membrane protein 1 (LMP1), the major oncoprotein produced by Epstein-Barr virus found in nasopharyngeal carcinoma (NPC), significantly elevated HIF1-α levels within exosomes; these exosomes retained transcriptional activity upon uptake by recipient cells (90).

### 4.2 Effects on angiogenesis

Angiogenesis is a crucial process in tumor growth and metastasis, and tumors might undergo an “angiogenic switch” to activate their vascular network when reaching a size limit of typically 1–2 mm^3^ (91). Tumors must trigger the angiogenic switch and expand their vascular network to surpass this restricted size and sustain indefinite proliferatio.

Angiogenesis is an inevitable outcome of the complex interplay between pro-angiogenic and anti-angiogenic factors. Under normal conditions, a delicate equilibrium exists between these opposing forces. However, this balance is disrupted during the early stages of tumor progression due to hypoxia, inflammation, and other precipitating factors. Hypoxia directly triggers the activation of numerous pro-angiogenic factors, such as VEGF and its receptors FLT-1 and FLK-1, via the HIF pathway. Additionally, plasminogen activator inhibitor-1 (PAI-1), angiopoietins (ANG-1 and ANG-2), and platelet-derived growth factor-B (PDGF-B) are also directly involved in tumor angiogenesis (92, 93).

In hypoxia-driven pro-angiogenic tumor responses, exosomes function as potent mediators facilitating hypoxia-dependent intercellular communication between malignant cells and vascular cells, such as endothelial cells and pericytes. miR-21-5p is significantly upregulated in the exosomes derived from hypoxia in thyroid papillary carcinoma BCPAP cells, which directly targets and inhibits TGFBI and COL4A1, enhancing angiogenesis in HUVEC (94). Renal cancer cells exposed to hypoxic stimulation secrete increased CA9-containing exosomes that bind to endothelial cells, promoting angiogenesis (95). The impact of exosomes on blood vessels extends beyond promoting angiogenesis. Lung cancer cell-derived exosome miR-23a under hypoxia inhibits the tight junction protein ZO-1, leading to increased vascular permeability and facilitating transendothelial migration during cancer progression (96).

### 4.3 Impact on tumor metastasis

During metastasis, a specific subset of circulating tumor cells, known as metastasis-initiating cells (MICs), plays a crucial role. These MICs reprogram distant cells by secreting exosomes, thereby transforming the distant microenvironment into one that is more conducive to tumor survival and growth, which in turn promotes tumor metastasis to distant sites (90). Consequently, exosomes function to optimize the microenvironment and establish pre-metastatic niches (PMNs) prior to the arrival of circulating tumor cells at these distant locations (97). In a mouse model of ductal adenocarcinoma (PDAC), PDAC releases exosomes highly expressing macrophage migration inhibitory factor (MIF), which are engulfed by Kupfer cells and transported from the bloodstream to the liver. This process induces the release of transforming growth factor β (TGFβ), promoting fibronectin production by hepatic stellate cells (hStCs). Fibronectin deposition further facilitates bone marrow-derived macrophages’ and neutrophils’ arrest within the liver, leading to PMN formation. Plasma exosomes isolated from patients with progressive PDAC exhibit significantly higher levels of MIF than those exhibited by exosomes isolated from patients without disease progression (98).

Studies have demonstrated that exosomes exhibit a propensity to target specific organs and tissues. This targeting facilitates the rapid establishment of pre-metastatic niches (PMNs) in these sites, thereby enhancing the metastatic potential of orthotopic tumors. Moreover, under hypoxic conditions, this tropism is further intensified by increased exosome secretion. In a hypoxic environment, colorectal cancer (CRC) cells can stimulate the release of exosomes rich in miR-135a-5p. Upon phagocytosis by KC cells, miR-135a-5p activates the large tumor inhibitory kinase 2-Yes-associated protein-matrix metalloproteinase 7 axis, leading to PMN formation and promoting liver metastasis in CRC (99). Lysyl oxidase-like 2 (LOXL2) is more abundant in hypoxic exosomes than it is in normoxic ones in head and neck squamous cell carcinoma cells. These LOXL2-rich exosomes are assimilated by distant fibroblasts and activate FAK/Src signaling in recipient fibroblasts, enhancing fibronectin production and recruiting myeloid-derived suppressor cells for PMN formation (100). Furthermore, studies on hypoxic exosomes (HypoExos) derived from prostate cancer (PCa) cells revealed elevated levels of metalloproteinases MMP2 and MMP9, extracellular matrix proteins such as fibronectin and collagen, and an increased number of CD11b+ cells at selective sites conducive for PMN formation (101). Therefore, the selection of target organs for tumor metastasis is not random; rather, it is facilitated by exosomes, which establish a fertile TME in these organs while delivering essential biomolecules.

### 4.4 Impact on immune evasion

Hypoxia induces the expression of diverse immunosuppressive molecules, including death receptor ligands (e.g., FasL or TRAIL), checkpoint receptor ligands (e.g., PD-L1), inhibitory cytokines (e.g., IL-10 and TGF-β1), prostaglandin E2 (PGE2), VEGF, and others, to suppress immune responses (102, 103). Exosomes carry immunosuppressive factors that can interfere with immune cell function and transfer them to host immune cells to block innate and adaptive immunity (104). PD-L1 and TGF-β carried by exosomes can interact with T cells, inhibit their proliferation, and suppress the immune response (105, 106). Under hypoxic conditions, miR-24-3p levels in nasopharyngeal carcinoma cell-derived exosomes increase. miR-24-3p inhibits T cell proliferation and Th1/Th17 differentiation while inducing regulatory T cell expansion (107).

Macrophages play a crucial role in tumor progression. Exosomes derived from hypoxic lung cancer cells can enhance the enrichment of miR-103a, augment the expression of M2-type cytokines and pro-angiogenic factors, and induce a phenotypic shift in macrophages towards pro-tumor activity (108). In hypoxic ovarian cancer (109) and pancreatic cancer (110), exosomes mediate the polarization of M2-type macrophages into immunosuppressive M2-like macrophages expressing PD-L1 and IL-10, inhibiting the proliferation of CD4+ and CD8+ T cells *in vitro*; *in vivo*, this process promotes tumor growth through PD-L1 (111). Natural killer group 2 member D (NKG2D), an activating receptor expressed on natural killer (NK) cells and CD56+ and CD8+T cells, plays a pivotal role in innate immunity. Hypoxia can transfer TGF-β1 to NK cells leading to reduced cell surface expression of activating receptor NKG2D, impairing NK cell function. More exosomes containing miR-23a are released, which target CD107a expression in NK cells, attenuating their cytotoxicity and resulting in ineffective eradication of cancer cells by NK cells (112).

## 5 Clinical application of the exosomes

Although exosomes under hypoxic conditions are involved in tumor cell signaling and promote tumor growth, they retain the advantages of normal exosomes, such as longevity, high stability, and specific targeting (113). In contemporary cancer therapy, many anti-tumor agents require crossing the cell membrane to exert their effects. However, due to poor water solubility, premature degradation, and high toxicity, these chemical drugs often fail to achieve optimal therapeutic outcomes and cause significant side effects (114). The stable bilayer lipid membrane of exosomes can effectively address these challenges. Consequently, numerous studies have utilized exosomes as drug carriers for tumor therapy, delivering them directly to target sites. Additionally, research has confirmed that exosomes can serve as efficient transporters to deliver cargo into tumor cells, demonstrating substantial potential in tumor therapy (**Table 1**).

**Table 1:** Exosomes can be used as vehicles for cancer therapy.

Cargo type	Exosome source	Cargo	Cancer type	Observations
Chemicals	Pancreatic cancer cells	Curcumin	Pancreatic cancer cells	Reducing viability of pancreatic cancer cells (115)
	Macrophages	Taxol	Metastatic Lung Cancer cells	Preferentially accumulated in cancer cells and inhibiting the growth of metastatic tumors (116)
	Mesenchymal stromal cells	Taxol	Pancreatic adenocarcinoma cells	Inhibiting tumor cell proliferation (117)
	Dendritic cells	Adriamycin	Breast cancer cells	Inhibiting tumor growth (118)
	Ginger root	Adriamycin	Colon cancer cells	Enhancing tumor growth inhibition (119)
miRNA	Mesenchymal stem cells	Anti-miR-9	Glioblastoma multiforme cells	Reversing expression of multidrug transporters and improving drug resistance (120)
	Mesenchymal stromal cells	MicroRNA-125b	Hepatocellular carcinoma	Decreasing HCC cell proliferation *in vitro* (121)
	Plasma	miR-101	Osteosarcoma cells	Inhibiting invasion and migration of osteosarcoma cells (122)
	Mesenchymal stem cells	miR-379	Breast cancer cells	Tumor formation and growth are significantly inhibited (123)
	Mesenchymal stem cells	miR-199a	Hepatocellular carcinoma	Significantly increasing the sensitivity of HCC cells to doxorubicin (124)
Other RNA	Mesenchymal cells	Kras^G12D^	Pancreatic cancer	Improving overall survival of mice (125)
	Bone marrow mesenchymal stem cells	GRP78 siRNA	Hepatocellular carcinoma	Sensitizing and reversing sorafenib-resistant cancer cells to sorafenib (126)

Many preclinical studies have evaluated the viability of exosomes as delivery vehicles for cancer treatment and highlighted their potential clinical significance. The heterogeneity of exosomes and their enrichment under hypoxic conditions make them promising noninvasive biomarkers for detecting tumor hypoxia. Sun et al. developed an HCC-specific extracellular vesicle (EV) purification system based on covalent chemistry, enabling early detection of hepatocellular carcinoma (HCC) with high sensitivity through digital scoring of purified EVs (127). Exosomes can be detected in nearly all body fluids at early stages, making them ideal biomarkers for monitoring dynamic intratumoral heterogeneity. This represents a novel noninvasive strategy for cancer diagnosis. Bjørnetrø et al. identified oxygen-sensitive miRNAs 486-5p, 181a-5p, and 30d-5p in EVs from colorectal cancer (CRC) cell lines in plasma samples, which were validated as circulating markers for high-risk locally advanced rectal cancer (LARC), demonstrating the feasibility of using exosomes as biomarkers (128).

## 6 Conclusions

Hypoxia is a distinctive and pervasive characteristic of the malignant tumor microenvironment (TME). Tumor cells release exosomes to transmit diverse signals that facilitate their survival in hypoxic conditions. This intercellular communication dynamically remodels the microenvironment through feedback mechanisms. Although the detailed regulatory mechanisms of exosomes remain largely elusive due to their complexity, existing evidence indicates that exosomes play a crucial role in tumor initiation and progression. A more comprehensive understanding of exosomes within hypoxic microenvironments could lead to significant advancements in cancer therapy. Hypoxia-induced exosomes hold considerable potential for both the detection and treatment of hypoxic tumors, and identifying new therapeutic targets may improve patient survival and prognosis.
